# Involvement of p63 in the *herpes simplex virus*-1-induced demise of corneal cells

**DOI:** 10.1186/1423-0127-17-47

**Published:** 2010-06-07

**Authors:** László Orosz, Éva Gallyas, Lajos Kemény, Yvette Mándi, Andrea Facskó, Klára Megyeri

**Affiliations:** 1 Department of Medical Microbiology and Immunobiology, University of Szeged, Dóm tér 10, H-6720 Szeged, Hungary; 2 Department of Ophthalmology, University of Szeged, Korányi fasor 10-11, H-6720 Szeged, Hungary; 3 Department of Dermatology and Allergology, University of Szeged, Korányi fasor 6, H-6720 Szeged, Hungary; 4 Dermatological Research Group of the Hungarian Academy of Sciences, Korányi fasor 6, H-6720 Szeged, Hungary; 5 Department of Ophthalmology, University of Debrecen, Nagyerdei körút 98, H-4032 Debrecen, Hungary

## Abstract

**Background:**

The transcription factor p63 plays a pivotal role in the development and maintenance of epithelial tissues, including the ocular surface. In an effort to gain insight into the pathogenesis of keratitis caused by HSV-1, we determined the expression patterns of the p63 and Bax proteins in the Staatens Seruminstitute Rabbit Cornea cell line (SIRC).

**Methods:**

SIRC cells were infected with HSV-1 at various multiplicities and maintained for different periods of time. Virus replication was measured by indirect immunofluorescence assay and Western blot analysis. Cell viability was determined by MTT assay. The apoptotic response of the infected cells was quantified by ELISA detecting the enrichment of nucleosomes in the cytoplasm. Western blot analysis was used to determine the levels of p63 and Bax proteins.

**Results:**

Indirect immunofluorescence assays and Western blot analyses demonstrated the presence of HSV-1 glycoprotein D (gD) in the infected SIRC cell line, and the pattern of gD expression was consistent with efficient viral replication. The results of MTT and ELISA assays showed that HSV-1 elicited a strong cytopathic effect, and apoptosis played an important role in the demise of the infected cells. Mock-infected SIRC cells displayed the constitutive expression of ΔNp63α. The expressions of the Bax-β and TAp63γ isoforms were considerably increased, whereas the level of ΔNp63α was decreased in the HSV-1-infected SIRC cells. Experiments involving the use of acyclovir showed that viral DNA replication was necessary for the accumulation of TAp63γ.

**Conclusion:**

These data suggest that a direct, virus-mediated cytopathic effect may play an important role in the pathogenic mechanism of herpetic keratitis. By disturbing the delicate balance between the pro-survival ΔN and the pro-apoptotic TA isoforms, HSV-1 may cause profound alterations in the viability of the ocular cells and in the tissue homeostasis of the ocular surface.

## Background

**Figure 1:**
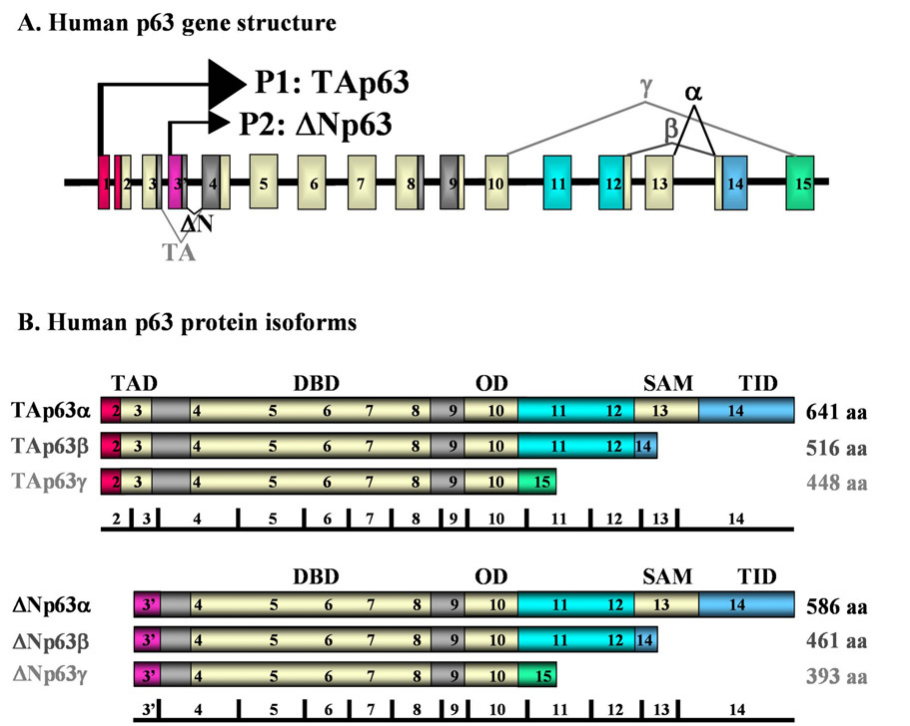
**(A) Gene architecture of human p63**. The alternative promoters and spicing events used to generate the various p63 isoforms are indicated. (B) Domain structure of the various p63 proteins. The transcription activation domain (TAD), DNA binding domain (DBD), oligomerisation domain (OD), sterile α motif (SAM) and the transinhibitor domain (TID) are depicted. The molecular size of each isoform is indicated on the right. (Not drawn to scale; adapted from [2-7]). aa, amino acid

The p53 family member p63 has been shown to play a pivotal role in the homeostatic renewal of epithelial tissues [1-3]. There are six p63 protein isoforms, which can be expressed from two different promoters, one immediately preceding the first exon and the second one lying in the third intron (Fig. 1) [1-8]. Transcription from the first and second promoters gives rise to TA- or ΔN-amino-termini of p63, respectively (Fig. 1) [1-8]. The TA isoforms possess an N-terminal acidic transactivation domain, while the ΔNp63 proteins lack this domain (Fig. 1) [1-8]. A great body of experimental evidence indicates that the TAp63 isoforms can induce cell death through a canonical p53-responsive DNA binding site [1-12]. In contrast, the ΔNp63 proteins can act in a dominant negative manner toward p53-mediated transcriptional activation [1-12]. Both TA and ΔN transcripts can undergo alternative splicing, leading to the formation of three C-terminal variants, denoted α, β and γ, which further increase the diversity of the p63 proteins (Fig. 1) [1-8]. Several interesting studies have clearly demonstrated that the ΔNp63α isoform plays an important role in the maintenance of the conjunctival and corneal stem cells, while ΔNp63β and ΔNp63γ contribute to the regulation of cell differentiation and regeneration in the conjunctiva, limbus and cornea [13-19]. Although the importance of p63 in the homeostasis of the ocular surface is widely accepted, the effects of infectious agents on the expression of this transcription factor family have not yet been investigated in epithelial cells of the eye.

Herpetic keratitis is a vision-threatening viral disease of the eye that is the major infectious cause of blindness in the developed countries [20-22]. The causative agent, Herpes simplex virus 1 (HSV-1) is a member of the *Herpesviridae *family comprising large, enveloped DNA viruses [23]. Primary herpetic keratitis can develop directly via 'front-door' route infection by droplet spread, or via a 'back-door' route, which involves the indirect spread of HSV-1 to the cornea from a non-ocular site [20]. HSV-1 infection may affect all three corneal layers, leading to epithelial, stromal and endothelial keratitis, respectively. Epithelial keratitis can be characterized by the appearance of branching dendritiform, or enlarged geographic ulcers [21]. Stromal keratitis and endothelitis can result in stromal scarring, thinning, neovascularization, severe iridocyclitis and an elevated intraocular pressure [20]. Most cases of corneal ulceration will eventually resolve, though recurrent infections impair the corneal function and lead to a vision impairment that may even necessitate penetrating keratoplasty. Previous studies have revealed that the mechanism of herpetic keratitis involves both immune- and virus-mediated cytopathogenic processes [24-28]. Whereas the immune processes involved in the pathogenesis of herpetic ocular surface diseases have been investigated extensively, the molecular events implicated in the direct cytopathic action of HSV-1 remain largely unknown.

In the present study, we examined the effects of HSV-1 on the expression of p63 and the Bcl-2 family member Bax in an effort to gain a better understanding of the ocular cytopathogenicity elicited by this virus.

## Methods

### Cell culture and HSV-1 growth

The Staatens Seruminstitute Rabbit Cornea (SIRC) cell line, was grown in Dulbecco's modified Eagle's minimal essential medium (Sigma Chemical Co., St. Louis, MO, USA) supplemented with 10% fetal calf serum (Gibco/BRL, Grand Island, NY, USA) at 37°C in a 5% CO_2 _atmosphere.

The KOS strain of HSV-1 was propagated at a multiplicity of infection (MOI) of 0.001 plaque-forming unit (PFU) per cell in Vero cell cultures for 3 days at 37°C. The culture fluid of HSV-1-infected Vero cells was harvested, quantified by plaque assay, stored at -70°C, and used as the infecting stock of the virus.

For experiments, SIRC cell cultures were inoculated with HSV-1 at different MOIs. 9-[(2-Hydroxyethoxy)methyl]guanine [Acyclovir (ACG); (Sigma)] was used at various concentrations when indicated. Every experiment was repeated at least three times.

### Indirect Immunofluorescence assay

Cytospin cell preparations were fixed in methanol-acetone (1:1) for 15 minutes (min) at -20°C. Slides were incubated with a 1:200 dilution of polyclonal rabbit anti-HSV glycoprotein D (gD) immunoglobulin (Sigma) for 1 h at 37°C. After washing with phosphate-buffered saline (PBS), the samples were reacted with fluorescein isothiocyanate-conjugated anti rabbit antibody (1:160) (Sigma) and incubated for 1 h at 37°C. After washing with PBS, the slides were visualized by confocal microscopy. The ratio of positive to negative cells was determined after counting 1,000 cells in random fields.

### Quantification of cell viability by MTT assay

The viability of HSV-1-infected cells was measured with the colorimetric MTT [3-(4,5-dimethylthiazol-2-yl)2,5-diphenyltetrazolium bromide] assay Tox-1 kit (Sigma). In this assay, SIRC cells were seeded in 96-well plates at a density of 1 × 10^4^/well. The cultures were infected with HSV-1 at different MOIs. At 48 h postinfection at 37°C, 10 μl MTT reagent (5 mg/ml) was added to each well. After 2 h incubation, MTT solvent containing 0.1 M HCl and isopropanol was added for 15 h. Absorbance was measured at 545 and 630 nm. The ratio of living cells was calculated via the following formula: percentage viability = [(absorbance of infected cells - blank)/(absorbance of corresponding mock-infected control cells - blank)] × 100.

### Quantification of apoptosis by enzyme-linked immunosorbent assay (ELISA)

The cells were washed in phosphate buffered saline (PBS) and the cell pellet was processed in a cell death detection ELISA kit (Roche Diagnostics GmbH, Penzberg, Germany) based on the measurement of histones complexed with mono- and oligonucleosome fragments formed during cell death. For this assay, the cells were incubated in lysis buffer for 30 minutes (min) and centrifuged at 12,000 rpm for 10 min. The supernatants were transferred into a streptavidin-coated microplate and incubated with biotin-conjugated anti-histone and peroxidase-conjugated anti-DNA monoclonal antibodies for 2 h. After washing, substrate solution 2,2'-azino-bis(3-ethylbenzthiazoline-6-sulphonic acid) (ABTS) was added to each well for 15 min. Absorbance was measured at 405 and 490 nm. The specific enrichment of mono- and oligonucleosomes was calculated as enrichment factor (EF) = absorbance of HSV-1-infected cells/absorbance of corresponding non-infected control cells.

### Western blot assays

Cells (1 × 10^7^) were homogenized in ice-cold lysis buffer containing 150 mM NaCl, 10 mM Tris HCl, pH 7.6, 5 mM EDTA, 1% (v/v) Nonidet P-40, 0.1% SDS, 1% sodium deoxycholate and protease inhibitor cocktail (Sigma), and the mixture was then centrifuged at 10,000 *g *for 10 min to remove cell debris. Protein concentrations of cell lysates were determined by using the Bio-Rad protein assay (Bio-Rad, Hercules, CA, USA). Supernatants were mixed with Laemmli's sample buffer and boiled for 3 min. Aliquots of the supernatants, containing 50 μg of total protein to detect p63, HSV D glycoprotein (gD) and Bax, were resolved by SDS-PAGE and electrotransferred onto nitrocellulose filters (Amersham, Buckinghamshire, UK). Preblocked blots were reacted with specific antibodies to HSV gD (Sigma), p63 detecting all of the various p63 isoforms (clone 4A4) (Santa Cruz Biotechnology Inc., Cambridge, MA, USA), p40 detecting the ΔNp63 isoforms (Merck KGaA, Darmstadt, Germany) and Bax (PharMingen, SanDiego, CA, USA) for 4 h in PBS containing 0.05% (v/v) Tween 20, 1% (w/v) dried non-fat milk (Difco Laboratories, Detroit, MI, USA) and 1% (w/v) BSA [fraction V; (Sigma)]. Blots were then incubated for 2 h with species-specific secondary antibodies coupled to peroxidase [peroxidase-conjugated anti-mouse antibody (DakoCytomation, Carpinteria, CA, USA), or peroxidase-conjugated anti-rabbit antibody (DakoCytomation)]. Filters were washed five times in PBS-Tween for 5 min after each step and were developed by using a chemiluminescence detection system (Amersham). The autoradiographs were scanned with a GS-800 densitometer (Bio-Rad), and the relative band intensities were quantified by use of the ImageQuant software (Amersham).

### Statistical analysis

All values are expressed as means ± standard deviation (SD). The one-way ANOVA test with the Bonferroni post-test was used for pairwise multiple comparisons, and *P *values < 0.05 were considered statistically significant.

## Results

### HSV-1-infected SIRC cells exhibit gD expression and increased apoptotic rates

The SIRC cell line was infected with the KOS strain of HSV-1 at various multiplicities and maintained for different periods of time.

**Figure 2:**
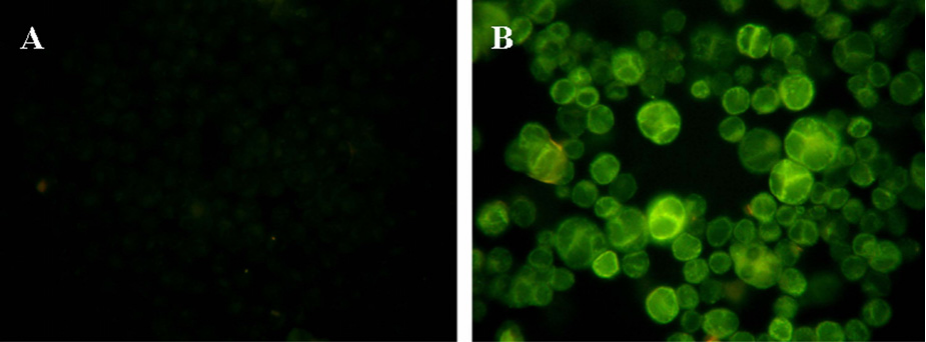
**Replication of HSV-1 in the SIRC cell line**. SIRC cells were infected with the KOS strain of HSV-1 at an MOI of 1 for 48 h (B). Mock-infected SIRC cells cultured in parallel were left untreated (A). HSV-1 replication was examined by confocal microscopy after staining with an HSV gD protein-specific rabbit polyclonal antibody preparation and FITC-conjugated anti-rabbit immunoglobulin. Results are representative of three independent experiments.

Indirect immunofluorescence assays to evaluate HSV-1 replication revealed positive staining for gD at 48 h postinfection (hpi) in ≥ 99% of SIRC cells infected at an MOI of 1 (Fig. 2).

**Figure 3:**
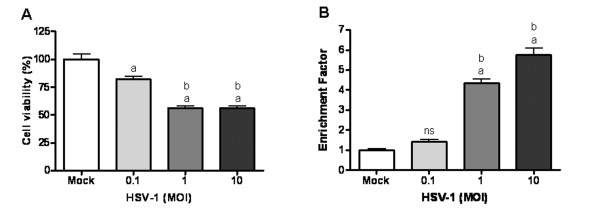
**HSV-1 induces cell death in the SIRC cell line**. SIRC cells were infected with HSV-1 at different MOIs for 48 h. Mock-infected cells cultured in parallel were left untreated. The cell viability was measured by using the MTT assay (A). Apoptosis was detected by measuring the specific enrichment of mono- and oligonucleosomes in the cytoplasm by ELISA (B). The enrichment factor was calculated as the absorbance of HSV-1-infected cells/absorbance of corresponding non-infected control cells. Data are mean (± SD) values from four independent experiments. *P *values were calculated by the ANOVA test with the Bonferroni post-test. ^a^*P *< 0.001 *vs. *mock; ^b^*P *< 0.001 *vs. *0.1 MOI; ^c^*P *< 0.001 *vs. *1 MOI; ns = nonsignificant *vs. *mock.

MTT assays to evaluate the cytopathogenicity of HSV-1 revealed decreased viability at 48 hpi in 23 and 36% of SIRC cells infected at MOIs of 1 and 10, respectively (Fig. 3).

ELISA to evaluate the extent of apoptosis revealed increased apoptotic rates in HSV-1-infected SIRC cells at 48 hpi; the EFs measured at MOIs of 0.1, 1 and 10 were 1.42, 4.35 and 5.8, respectively (Fig. 3).

Together, these data demonstrate the expression of HSV-1 gD protein that is consistent with efficient viral replication. Moreover, these results reveal that HSV-1 elicits a strong cytopathic effect in the SIRC cell line, and apoptosis plays an important role in the demise of the infected cells.

### HSV-1 alters the levels of Bax and p63 proteins

To determine whether HSV-1 can alter the expressions of Bax and p63, the steady-state levels of these proteins were determined by Western blot analysis.

**Figure 4:**
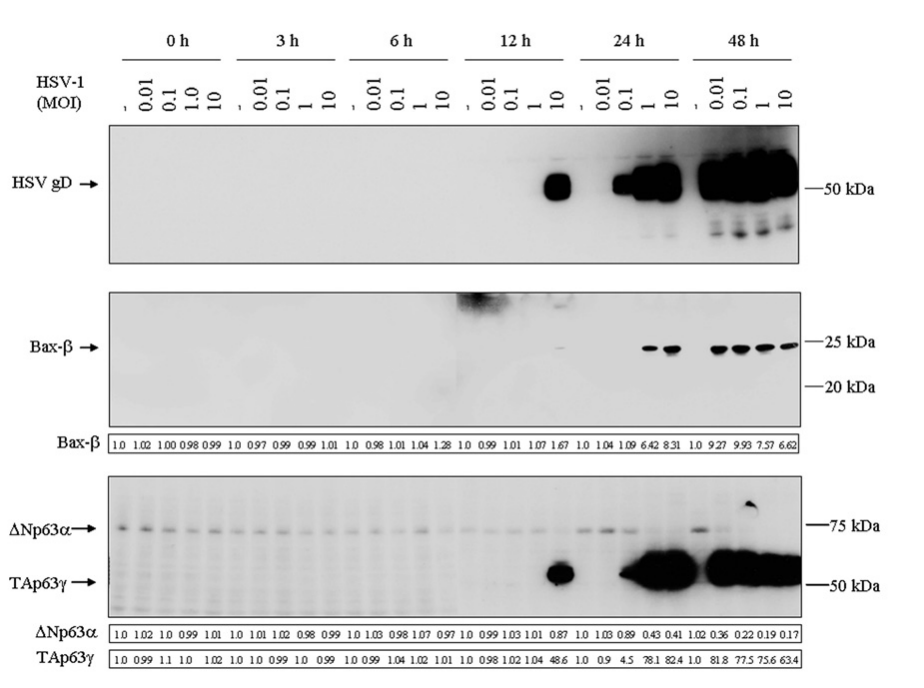
**HSV-1 infection alters the levels of p63 and Bax-β in the SIRC cell line**. Total protein was isolated from mock-infected cells and from cultures infected with HSV-1 at MOIs of 0.001, 0.01, 0.1, 1 and 10 at the indicated time points. Samples were resolved on SDS-PAGE and transferred onto nitrocellulose filters. The steady-state levels of gD, Bax-α, Bax-β and p63 were analyzed by Western blot assay. To determine protein levels in HSV-1-infected cells, band intensities were quantified by use of the ImageQuant software. The numbers indicate the relative quantities of each band, normalized to the control cells at each time point. Lanes 1, 6, 11, 16, 21 and 26, mock-infected cells; lanes 2-5, 7-10, 12-15, 17-20, 22-25 and 27-30, HSV-1-infected cultures. The results are representative of three independent experiments.

First, the kinetics of HSV-1 gD expression was investigated. The presence of gD was observed in the SIRC cell cultures infected with HSV-1 at an MOI of 10 at 12 hpi (Fig. 4; lane 20). The gD protein accumulated in the cultures infected with HSV-1 at MOIs of 0.1, 1 and 10 at 24 hpi (Fig. 4; lanes 23-25). High-level expression of the gD protein was also revealed in every culture infected with HSV-1 by 48 hpi (Fig. 4; lanes 27-30).

The analysis revealed the presence of a Bax isoform corresponding to Bax-β in HSV-1-infected SIRC cultures at 12 hpi (the relative quantity of Bax-β in cells infected at an MOI of 10 was 1.67) (Fig. 4; lane 20). At the 24-h time point, the expression of the Bax-β protein in the HSV-1-infected SIRC cultures was upregulated (the relative quantities of Bax-β in cells infected at MOIs of 1 and 10 were 6.42 and 8.31, respectively) (Fig. 4; lanes 24 and 25). At the 48-h time point, the HSV-1-infected SIRC cultures displayed elevated levels of Bax-β (the relative quantities of Bax-β in cells infected at MOIs of 0.01, 0.1, 1 and 10 were 9.27, 9.93, 7.57 and 6.62, respectively) (Fig. 4; lanes 27-30).

The expression pattern of p63 was determined by using an antibody preparation which recognizes all of the various p63 isoforms. The analysis revealed the constitutive expression of a p63 protein migrating near 68 kDa in the mock-infected SIRC cells (lanes 1, 6, 11, 16, 21 and 26 in Fig. 4). Previously published data demonstrated that the 68 kDa protein possibly corresponds to ΔNp63α [4]. At 12 hpi, the expression of ΔNp63α in the HSV-1-infected SIRC cultures was downregulated (the relative quantity of ΔNp63α in cells infected at an MOI of 10 was 0.87) (Fig. 4; lane 20). At the 24-h time point, HSV-1 triggered an impressive reduction in the level of ΔNp63α in the SIRC cells (the relative quantities in cells infected at MOIs of 0.01, 0.1, 1 and 10 were 0.89, 0.43 and 0.41, respectively) (Fig. 4; lanes 23-25). At the 48-h time point, the HSV-1-infected SIRC cultures exhibited decreased levels of ΔNp63α (the relative quantities in cells infected at MOIs of 0.01, 0.1, 1 and 10 were 0.36, 0.22, 0.19 and 0.17, respectively) (Fig. 4; lanes 27-30).

The experiments also revealed the presence of a 51-62 kDa protein in HSV-1-infected SIRC cultures. Previously published data demonstrated that the 51-62 kDa protein possibly corresponds to TAp63γ [4]. At 12 hpi, HSV-1-infected SIRC cells exhibited increased levels of TAp63γ (the relative quantity of TAp63γ in cells infected at an MOI of 10 was 48.6) (Fig. 4; lane 20). At the 24-h time point, the expression of TAp63γ in the HSV-1-infected SIRC cultures was highly upregulated (the relative quantities in cells infected at MOIs of 0.1, 1 and 10 were 4.5, 78.1 and 82.4) (Fig. 4; lanes 23-25). At 48-h postinfection, the HSV-1-infected SIRC cultures displayed elevated levels of TAp63γ (the relative quantities in cells infected at MOIs of 0.01, 0.1, 1 and 10 were 81.8, 77.5, 75.6 and 63.4, respectively) (Fig. 4; lanes 27-30).

**Figure 5:**
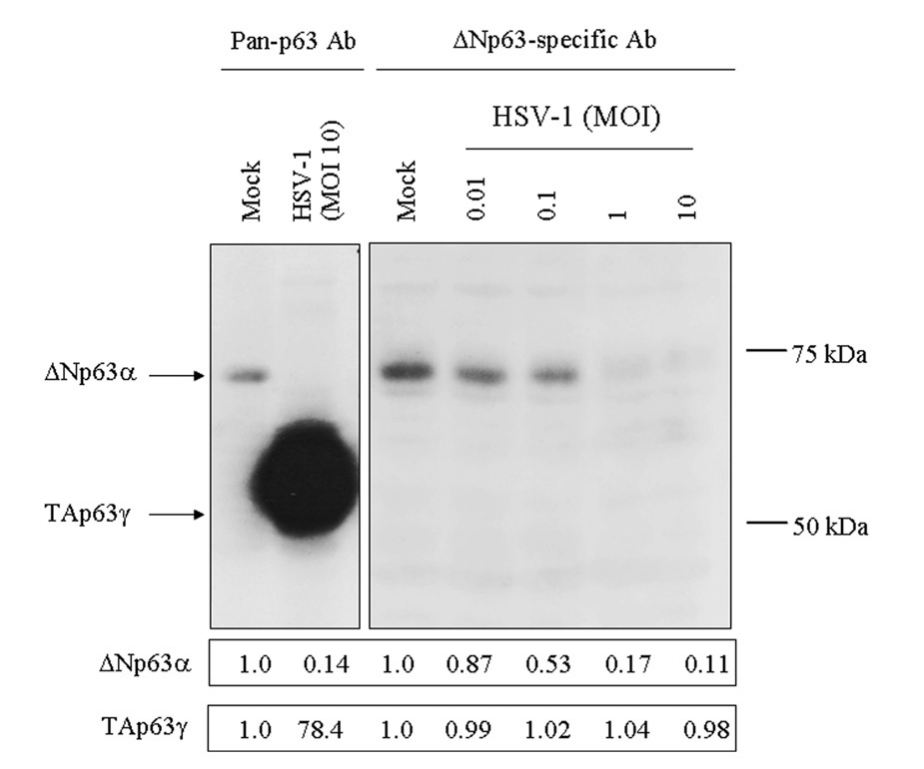
**Serological identification of the p63 isoforms expressed in HSV-1-infected SIRC cells**. The levels of different p63 isoforms were detected at 24 hpi in mock-infected and HSV-1-infected SIRC cells by Western blot analysis, using an antibody preparation that recognizes all of the various p63 isoforms (lanes 1 and 2) and a ΔN-isoform-specific immunoglobulin (lanes 3-7).

To identify the p63 isoforms, the steady-state levels of these proteins were determined by Western blot analysis, using a polyclonal antiserum which reacts only with the ΔN forms. The ΔNp63-specific antibody preparation detected the 68 kDa p63 isoform in the mock-infected SIRC cells, but failed to recognize the 51-62 kDa p63 isoform in the cultures infected with HSV-1 at an MOI of 10 for 24 hpi (Fig 5). These results clearly reveal that the 68 kDa p63 protein detected in the mock-infected SIRC cells is ΔNp63α, while the 51-62 kDa p63 isoform detected in HSV-1-infected cultures is TAp63γ.

Together, these results indicate that HSV-1 modulates the expression patterns of Bax and p63. The level of ΔNp63α was decreased, while the expressions of Bax-β and TAp63γ were highly increased in the HSV-1-infected SIRC cells.

### HSV-1-mediated TAp63γ expression requires viral DNA replication

**Figure 6:**
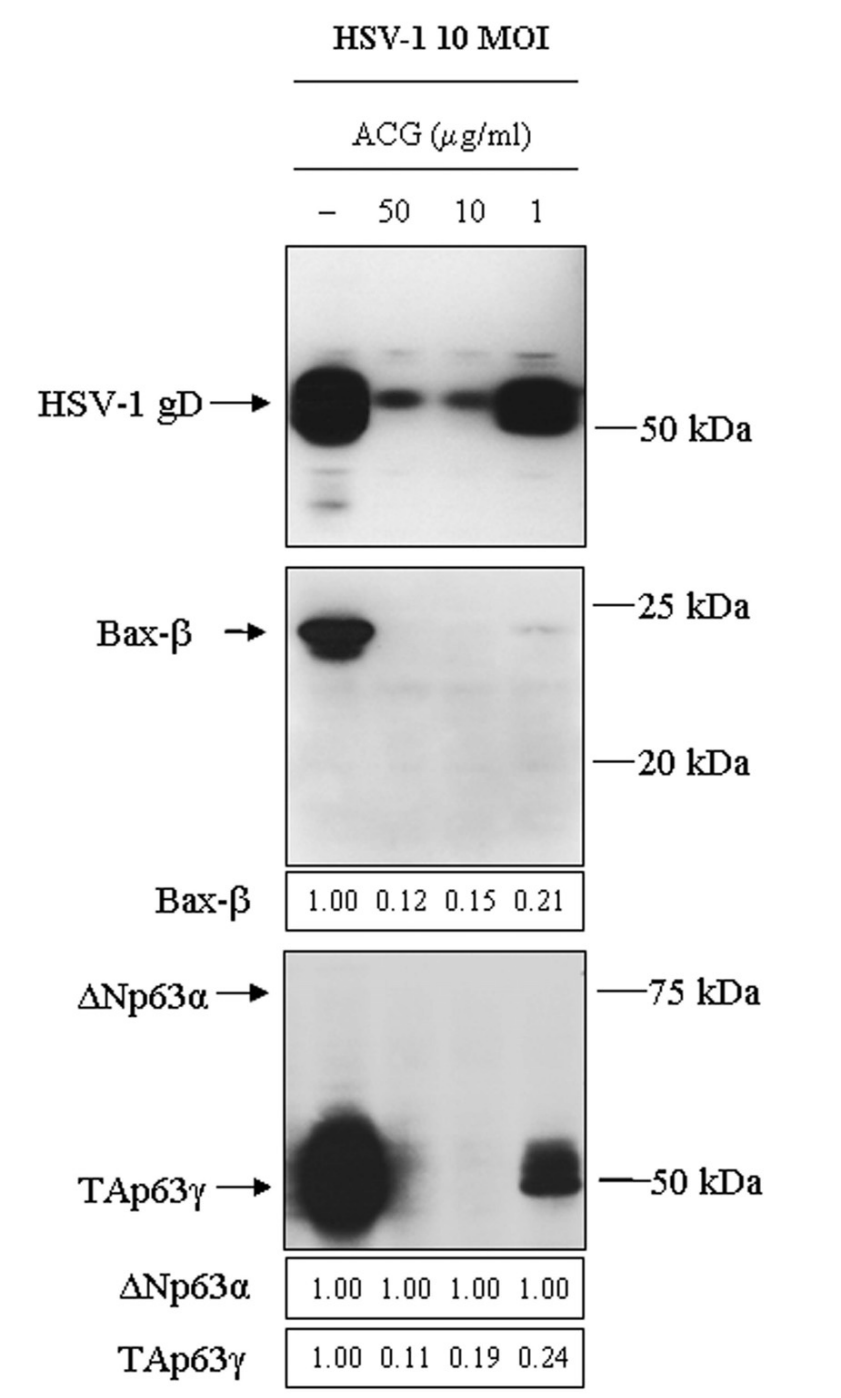
**The HSV-1-mediated TAp63γ expression requires viral DNA replication**. SIRC cells were infected with the KOS strain of HSV-1 at an MOI of 10 and maintained for 24 h in the absence or in the presence of Acyclovir (ACG). To determine the dependence of the TAp63γ expression on HSV-1 DNA replication, the levels of gD, Bax and p63 were determined by Western blot assay. To determine protein levels in HSV-1-infected cells, band intensities were quantified by densitometric analysis with the Imagequant software. The numbers indicate the relative quantities of each band, normalized to the control cells at each time point. Lane 1: HSV-1-infected cells incubated in the absence of ACG; lanes 2-4: HSV-1-infected cells incubated in the presence of ACG. The results are representative of three independent experiments.

To investigate the basis of the HSV-1-induced increase of the TAp63γ level, SIRC cells were infected in the presence or absence of the viral DNA replication inhibitor ACG. The cells were analyzed for the presence of HSV gD, ΔNp63α, TAp63γ and Bax-β. The low level of the late protein gD expression in SIRC samples treated with 50 or 10 μg/ml ACG indicated that the drug treatment efficiently inhibited viral DNA replication (Fig. 6; lanes 2 and 3).

The Bax-β protein levels in the HSV-1-infected SIRC cells treated with 50, 10 and 1 μg/ml ACG were greatly decreased (the relative quantities of Bax-β in cells infected at an MOI of 10 were 0.12, 0.15 and 0.21, respectively) (Fig. 6; lanes 2-4).

The TAp63γ protein levels in the HSV-1-infected SIRC cells treated with 50 and 10 μg/ml ACG were greatly decreased (the relative quantities of TAp63γ in cells infected at an MOI of 10 were 0.11 and 0.19) (Fig. 6; lanes 2 and 3). The expression of the TAp63γ isoform in the HSV-1-infected cultures treated with 1 μg/ml ACG was downregulated (the relative quantity of TAp63γ in SIRC cells infected at an MOI of 10 was 0.24) (Fig. 6; lane 4).

## Discussion

This study, aiming to evaluate the role of p63 in the pathogenic mechanisms of herpetic ocular surface disease, revealed the presence of HSV-1 gD protein and a strong cytopathic effect in the HSV-1-infected rabbit corneal cell line (SIRC) (Figs 2, 3, 4). Our data have also indicated that apoptosis plays an important role in the demise of SIRC cells infected with HSV-1 (Fig. 3). These data are in full agreement with previous findings demonstrating that HSV-1 has the potential to elicit various forms of cell death, including necrosis, apoptosis, anoikis and autophagy [29-35].

Compelling evidence has accumulated that the various p63 isoforms play pivotal roles in several physiological and pathological processes of the ocular surface [13-19]. The ΔN and TA p63 subclasses operate in a concerted fashion to maintain the proliferative potential of the ocular surface epithelia and to control the processes of differentiation and regeneration in the conjunctiva and cornea [15,19]. The ocular surface may be exposed to harmful environmental stimuli, such as ultraviolet exposure, and may also function as an entry site for a wide array of human pathogenic microorganisms. By disturbing the delicate balance between the pro-survival ΔN and the pro-apoptotic TA isoforms, stress signals that alter the expression of p63 may cause profound alterations in the viability of the ocular cells and in the tissue homeostasis of the ocular surface. As a step in our investigations of the underlying molecular events implicated in HSV-1-induced ocular cytopathogenicity, we focused on the role of p63 in the SIRC cell line. Our experiments revealed the constitutive expression of ΔNp63α in the mock-infected SIRC cells (Fig. 4). Interestingly, we observed an impressive reduction in the level of the ΔNp63α and a dramatic rise in the level of TAp63γ following infection with HSV-1 (Fig. 4). The kinetics of HSV-1 replication and the level of TAp63γ expression correlated strictly (Fig. 4). Noteworthy previous studies raise the possibility that HSV-1 may alter the expression of p63 via multiple mechanisms [36-45]. Certain viral proteins may have the potential to alter the transcription of p63 or to affect the stability and activity of the p63 isoforms via the induction of their posttranslational modifications [36-44]. The virion-associated host shutoff protein [(vhs), also known as UL41], which causes the degradation of cellular and viral RNA [36,37], may evoke a decrease in the level of ΔNp63α mRNA. The α-trans-inducing factor [(α-TIF), also known as VP16 or UL48], which stimulates the transcription of IE genes via cellular transcription factors, such as the POU homeodomain protein Oct-1 (where Oct stands for octamer binding protein) and the host cell factor [38-40], may elicit an increase in the level of TAp63γ. The infected cell protein (ICP) 0, which controls the stability of cellular proteins and leads to the disruption of promyelocytic leukemia (PML) nuclear bodies [also known as PODs (PML oncogenic domains) and ND10 (nuclear domain 10)] [41-44], may dysregulate the expression pattern of p63. However, interesting studies have demonstrated that the replication of HSV-1 DNA activates the ataxia teleangiectasia mutated (ATM)-dependent signaling pathway implicated in the cellular DNA damage response (DDR) [45]. Since TAp63 isoforms have been shown to operate as important downstream mediators of DDR [46-48], it is conceivable that the dysregulation of p63 expression observed in HSV-1 infected SIRC cells is a result of the activation of DDR evoked by viral replication. Our experiments have shown that the viral DNA replication inhibitor ACG completely abolished the HSV-1-mediated induction of TAp63γ in SIRC cells, indicating that replication of viral DNA is necessary for the accumulation of TAp63γ (Fig. 6). This observation strongly supports the view that the dysregulation of p63 expression depends on the cellular DDR, but does not exclude the role of HSV-1-encoded proteins. Thus, additional studies are required to elucidate the potential contributions of vhs, α-TIF, ICP0 and other viral proteins to the development of the HSV-1-mediated dysregulation of p63 expression. Our data further demonstrated that HSV-1-infected SIRC cells display decreased viability and an increased apoptotic rate (Fig. 3). Together, these results suggest that the altered pattern of p63 expression observed in HSV-1-infected SIRC cells may represent a mechanism by which this virus perturbs the functions of the corneal epithelial cells and leads to their demise.

In line with these data, we next investigated the expression of Bax, which is known to be upregulated by TAp63α and TAp63γ [10,11]. Previous studies have demonstrated the existence of several Bax isoforms [49]. It is well documented that Bax-α is a central component of apoptosis induction [50]. In response to apoptotic stimuli, Bax-α becomes activated, translocates to the mitochondria and triggers the release of cytochrome *c *and caspase-9, which in turn results in the irreversible execution of the apoptotic program [51]. It has been reported that the Bax-β protein is expressed constitutively in several human cell types, and its level is controlled by proteasomal degradation [52]. Various stressors inhibit ubiquitination of the Bax-β protein and thereby prevent its proteasomal degradation, leading to the accumulation of this Bax isoform [52]. Similarly to Bax-α, Bax-β has the capability to trigger apoptosis via the mitochondrial pathway [52,53]. Moreover, Bax-β associates with and promotes Bax-α activation [53]. Our experiments revealed no constitutive expression of any of the Bax isoforms in the mock-infected SIRC cells (Fig. 4). Interestingly, we observed a dramatic rise in the level of Bax-β in HSV-1-infected cultures (Fig. 4). Following the demonstration of an altered Bax expression pattern in SIRC cells, we postulate an important role for Bax-β in the apoptotic responsiveness of corneal epithelial cells infected with HSV-1. Other interesting recent data have proved that HSVs encode ubiquitinating and deubiquitinating enzymes, which can modify the ubiquitination status of both viral and host cell proteins [54,55]. In view of these observations, it is reasonable to infer that the Bax-β protein may be a novel target of HSV-1-mediated deubiquitinating events. However, the precise molecular mechanisms responsible for stabilization of the Bax-β protein in HSV-1-infected cells remain to be elucidated.

## Conclusions

Overall, this study demonstrates that the KOS strain of HSV-1 modulates the patterns of p63 and Bax expression in the SIRC cell line. These data may bear on the pathogenic mechanisms of ocular diseases caused by HSV-1, as p63 and Bax isoforms play a pivotal role in the maintenance of the ocular surface integrity.

## Competing interests

The authors declare that they have no competing interests.

## Authors' contributions

LO designed and performed most experiments, and drafted the manuscript, ÉG helped to design experiments and edited the manuscript, LK helped to design experiments, interpreted the results and revised the manuscript, YM helped to design the experiments, interpreted the results and revised the manuscript, AF helped to design experiments and edited the manuscript, KM conceived of the study, performed research and revised the manuscript. All authors read and approved the final manuscript.
